# Electromagnetic Detection of a Perfect Carpet Cloak

**DOI:** 10.1038/srep10401

**Published:** 2015-05-22

**Authors:** Xihang Shi, Fei Gao, Xiao Lin, Baile Zhang

**Affiliations:** 1 Division of Physics and Applied Physics, School of Physical and Mathematical Sciences, Nanyang Technological University, 21 Nanyang Link, Singapore 637371, Singapore; 2 Centre for Disruptive Photonic Technologies, Nanyang Technological University, 21 Nanyang Link, Singapore 637371, Singapore

## Abstract

It has been shown that a spherical invisibility cloak originally proposed by Pendry *et al.* can be electromagnetically detected by shooting a charged particle through it, whose underlying mechanism stems from the asymmetry of transformation optics applied to motions of photons and charges [PRL 103, 243901 (2009)]. However, the conceptual three-dimensional invisibility cloak that exactly follows specifications of transformation optics is formidably difficult to implement, while the simplified cylindrical cloak that has been experimentally realized is inherently visible. On the other hand, the recent carpet cloak model has acquired remarkable experimental development, including a recently demonstrated full-parameter carpet cloak without any approximation in the required constitutive parameters. In this paper, we numerically investigate the electromagnetic radiation from a charged particle passing through a perfect carpet cloak and propose an experimentally verifiable model to demonstrate symmetry breaking of transformation optics.

Invisibility cloaking has been proposed as a representative application of transformation optics (TO)^1^,^2^—a geometrical optical design strategy which merges Einstein’s general relativity and Maxwell’s electromagnetism principles—to guide light around a hidden object in an effectively transformed electromagnetic (EM) space. The underlying mechanism of TO stems from the symmetry, or ‘form invariance,’ of Maxwell’s equations under a coordinate transformation, which thus enables creating a bent EM space that is equivalent to an empty EM space. A photon in the transformed EM space cannot judge if the EM space is transformed or not, and therefore will be deceived as if it were still in the empty EM space. While there are many ways to detect the cloak such as touching it by hands, the real challenge is how to make an invisibility cloak visible to the eyes. Recently, it has been pointed out that the symmetry of coordinate transformation only applies to photons, but not to motions of charged particles, although the latter are also inside the EM space^3^. The reason is that the behavior of photons is fully governed by Maxwell’s equations, and thus they perceive only the EM space. In contrast, a charged particle perceives not only the EM space, but also the mechanical space, where Newton’s laws dictate. Therefore, by referring to a flat mechanical space, a charged particle can easily tell if the EM space is transformed or not. In other words, a fast-moving charged particle can reveal the asymmetry of transformation optics.

This asymmetry has been utilized to propose an approach of detecting a perfect spherical cloak electromagnetically^3^. However, the spherical cloak with exact specifications of TO requires extreme values in constitutive parameters, being formidably difficult to implement. The experimentally realized cylindrical cloak^4^ utilized simplified parameters and thus is inherently visible^5^. On the other hand, the ‘carpet cloak,’ originally proposed in 2008 to hide a bump on a flat ground plane^6^,^7^, has trigged a boom of experimental studies^8^,^9^,^10^,^11^,^12^ because of its superior properties in terms of broad bandwidth and experimental feasibility. Further effort in this direction has pushed the carpet cloaking technology into visible spectrum to hide macroscopic objects^13^,^14^. Recently, a remarkable progress has been made^15^: a full-parameter cloak comprising of two carpet cloaks without approximation in constitutive parameters successfully cloaked an electrically large object in reality. This inspires us to investigate the radiation of a fast-moving charged particle through a perfect carpet cloak as an experimentally realizable model to demonstrate the asymmetry of transformation optics.

## Results

### Modelling of electron radiation through a perfect carpet cloak

**Figure 1:**
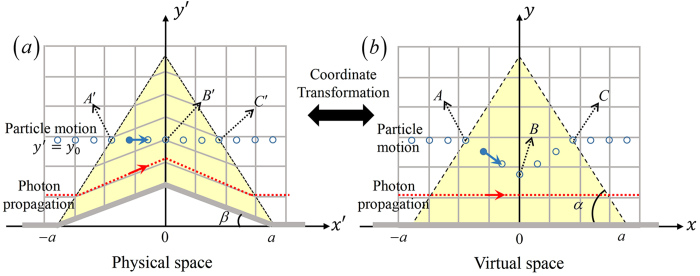
Trajectory of a fast-moving charged particle compared with that of a photon passing through (a) the carpet cloak and (b) its corresponding virtual space. **** The mesh in the background represents the EM space.

For simplicity, we perform derivation, computation, and experimental design in a two-dimensional (2D) geometry where all physical quantities are invariant along the third dimension. Fig. 1 shows a 2D carpet cloak and its corresponding virtual space. This carpet cloak is equivalent to a bent EM space that is transformed from the original flat EM space via the following coordinate transformation


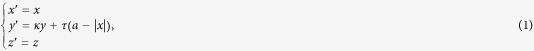


where 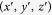 and 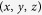 are the coordinates in the physical space and virtual space respectively, 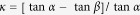 and 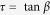. According to TO, the relative permittivity and permeability of the cloak can be obtained as


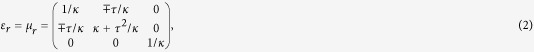


where the minus (positive) sign applies to the region of 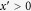 (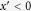).

In this 2D geometry, a photon that goes through the cloak in the physical space will follow a bent path as in Fig. 1a. Correspondingly, the path for the photon in the virtual space is a straight line as in Fig. 1b. The photon cannot judge if it is in the empty EM space before transformation or in the bent EM space that is transformed, as a result of the symmetry of coordinate transformation applied to photons. To demonstrate the asymmetry of coordinate transformation applied to charged particles, here we let a 2D line charged particle with charge 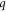 pass through the cloak with a uniform velocity 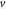 along a predetermined trajectory 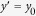. The charged particle will impinge on the cloak at point A’ in Fig. 1a, cross the middle point B’, and then leave the cloak at point C’. Correspondingly, in the virtual space it follows the trajectory 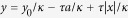. Its velocity in the virtual space increases by a factor of 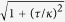
as the particle travels for a longer distance in the virtual space within the same time interval as that in the physical space. The motion of the charged particle along the bent trajectory in the virtual space will induce radiation that is detectable in the physical space. We first find the radiation emitted by the particle in the virtual space, and then transform the radiation from the virtual space to the physical space.

In the physical space, the current density of the fast-moving 2D line charged particle can be expressed as


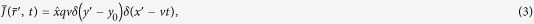


To avoid potential confusion, we will use the upper-case 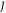 and lower-case 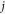 to represent current densities in the physical space and virtual space, respectively. The particle locates at 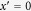 when 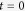. In the frequency domain, each spectral component of the current can be expressed as


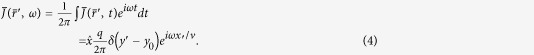


It will excite the following magnetic field in a homogeneous medium with permittivity 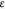 and permeability 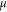,


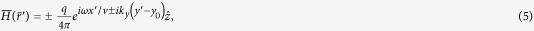


where the positive (negative) sign applies to the region of 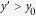 (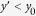) and 
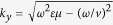
. In the virtual space, the current density in the segments AB and BC can be obtained through coordinate transformation:


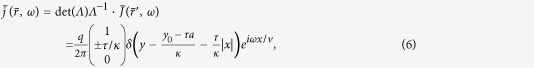


where the positive (negative) sign applies to 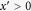 (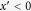) and 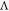 is the Jacobian transformation matrix.

Because the trajectory of the current in the virtual space is bent, to facilitate analysis, we cut each finite current segment (i.e. AB, BC and hypothetical AC) into small pieces and use a finite dipole array with a uniform phase difference 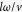 between adjacent dipoles separated with horizontal distance 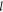 to model each finite current segment. The current density of the dipole array is the summation of all the dipoles. Therefore, a discretized version of the current density in equation (4) for the hypothetical segment AC in Fig. 1b is


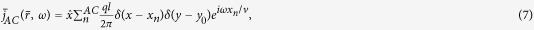


where 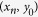 are the coordinates of the dipoles along segment AC. Correspondingly, the discretized version of the current density in equation (6) for segments AB and BC in Fig. 1b is


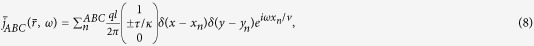


where 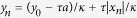, 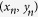 are the coordinates of the dipoles along segments AB and BC. The positive (negative) sign applies to the region of 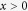 (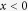). The magnetic field generated by the dipole array for the hypothetical segment AC in Fig. 1b is


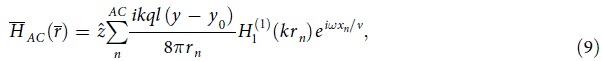


where 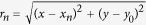 and 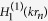 is the first order Hankel function of the first kind. Correspondingly, the magnetic field generated by the dipole arrays of segments AB and BC in Fig. 1b is


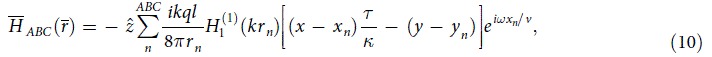


where 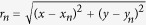.

To summarize, the current density in the virtual space in Fig. 1b can be expressed as


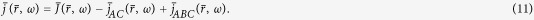


Reflection of the fields at the ground plane can be taken into account by making use of equivalent image sources. The current densities of the image sources of the fast-moving 2D line charged particle as well as the dipole arrays for corresponding current segments are


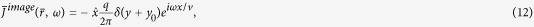



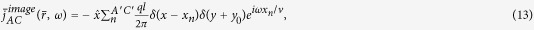



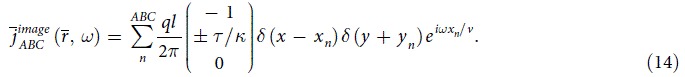


By labeling the fields generated by these image sources as 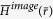, 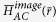 and 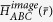 respectively, we can write down the total field in the virtual space for each spectral component as


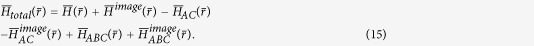


The field inside the cloak in the physical space can be obtained by applying coordinate transformation to the field in the virtual space. In the numerical modeling of the particle, we use a Gaussian profile 
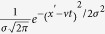
 to replace the delta function 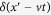 in equation (3). When 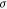 goes to zero, the Gaussian profile returns to the delta function. In the simulation, 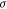 is chosen to be 0.1 µm which corresponds to a maximum bandwidth up to about 1000 THz. Before proceeding to discuss radiation from the charged particle with different velocities, we would like to clarify the reason of using the 2D line charged particle instead of a point one. From the above calculation, the field generated from the 2D line charged particle is transverse magnetic (TM) field. The full carpet cloak designed before works only with either transverse electric (TE) field or TM field^13^,^14^,^15^. By letting the radiation from the carpet cloak be TE field, we reveal the essence of the radiation-steming from the asymmetry of transformation optics applied to motions of photons and charges. In the experiment, the 2D line charged particle can be realized with a group of particles forming a line in the z direction.

### Numerical results of electron radiation through a perfect carpet cloak

To fix the parameters of the cloak model in simulation, we set the background here to be 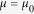 and 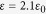, where 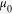 and 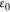 are the constitutive parameters of vacuum. We choose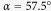 and 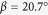, being similar to the geometry adopted in^13^,^14^. The length of the bottom of the cloak is 2a = 16µm Note that the resultant cloak possesses constitutive parameters all larger than one. The prime of the coordinates in the physical space will be omitted in the following for conciseness consideration.

**Figure 2:**
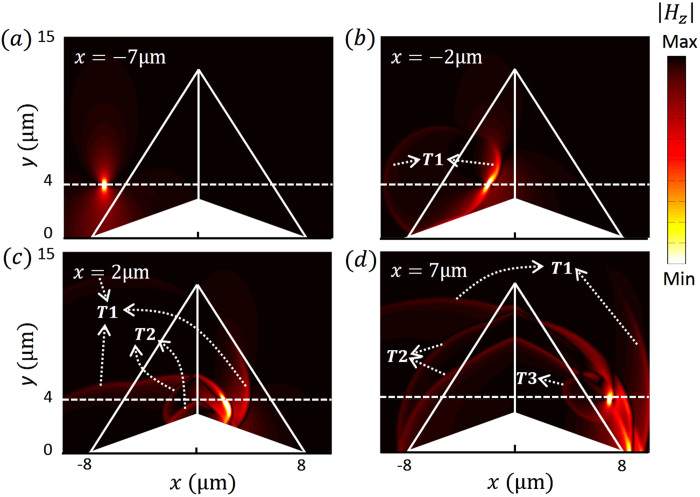
The magnetic field of radiation emitted by a charged particle passing through the carpet cloak with velocity 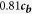, where 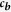 is the phase velocity of light in the background. The particle moves to different positions from a-d. T1, T2 and T3 are the emitted transition radiation.

**Figure 3:**
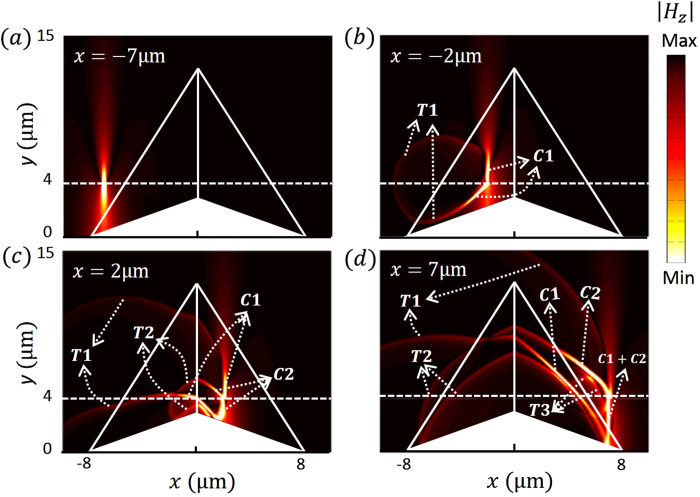
The magnetic field of radiation emitted by a charged particle passing through the carpet cloak with velocity 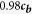, where 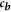 is the phase velocity of light in the background. The particle moves to different positions in a-d. T1, T2 and T3 are the emitted transition radiation. C1 and C2 are the Cherenkov radiation emitted by the charged particle inside the carpet cloak.

Since the trajectory of the particle in the virtual space is bent while the time remains the same as that in the physical space, the speed in the virtual space increases by a factor of 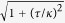 when the particle moves horizontally in the physical space. The virtual space is isotropic and homogeneous and Cherenkov radiation (CR) will be excited if the velocity of a charged particle is greater than the phase velocity 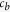 of light in the background. This means that in the physical space, a horizontally moving charged particle will excite CR when its velocity is greater than 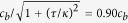. With reference to the phase velocity 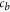 of light in the background, we choose two different velocities for the charged particle, 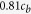 and 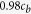 in the physical space, to investigate the radiation generated along the trajectory of the particle. The results are shown in Fig. 2 and Fig. 3 respectively. In both figures, the particle moves along the same trajectory 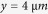. The particle’s motion can be divided into four stages: approaching the cloak, crossing the first interface between the cloak and the background, crossing the interface between the left and right parts of the cloak and finally leaving the cloak. In Fig. 2a, the particle is just approaching the cloak and there is no radiation. When it enters the cloak, a radiation labeled as T1 is emitted and forms a closed loop, which means the radiation propagates in all directions in the plane. This radiation corresponds to the sudden velocity change at point A in the virtual space (Fig. 1b), and also corresponds to transition radiation (TR) when the charged particle impinges on the boundary of the cloak at point A’ in the physical space (Fig. 1a). As shown in Fig. 2c, when the particle crosses the middle line separating the left and right parts of the cloak located at x=0 µm another TR, labeled as T2, is excited and forms a heart shape with a smaller heart shape embedded inside (the latter formed by the reflection at the bottom). It is worth mentioning that the left and right parts of the cloak are always impedance matched, even in previous experimentally realized imperfect models^13^,^14^. Therefore, photons will just go through this interface as if it were not there. However, for a charged particle, this interface still causes TR which corresponds to the sudden velocity change at point B in the virtual space (Fig. 1b). As the particle leaves the cloak, as shown in Fig. 2d, it excites another TR at the back interface of the cloak, labeled as T3, which forms another closed loop. This radiation apparently corresponds to the sudden velocity change at point C in the virtual space in Fig. 1b.

The above analysis of the charged particle shows TR from the charged particle when its velocity is relatively low. When the velocity of the charged particle is sufficiently high, another type of radiation, CR will be involved. Here, we choose the velocity of the particle to be 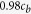. In Fig. 3a, the particle is moving toward the cloak without radiation. Note that because of its larger velocity, its associated field pattern is more squeezed horizontally as a result of Lorentz contraction. In Fig. 3b, the particle has entered the cloak. Besides the TR (T1) that is similar to Fig. 2b, the particle is generating CR (C1) that is like a cone shape but being asymmetry because of the anisotropy of the cloak. In Fig. 3c, the particle has crossed the middle line of the cloak. The newly generated TR (T2) is again like a heart shape, being similar to Fig. 2c. The lower branch of the CR (C1) in Fig. 3b now is reflected from the ground, while its upper branch is almost fully transmitted into the right part of the cloak. The newly generated CR (C2) now is partially overlapping with the transmitted CR (C1). In Fig. 3d, the particle has left the cloak. One can see that the transmitted upper branch of C1 and the transmitted lower branch of C2 form a strong radiation near the forward direction. The reflected lower branch of C1 and the transmitted upper branch of C2 form another strong radiation around the upper-right direction. The radiation excited is the combination of CR and TR.We emphasize that this radiation is entirely unrelated to the bremsstrahlung radiation when the charged particle impacts an atomic nucleus.

### Model design for experimental verification

**Figure 4:**
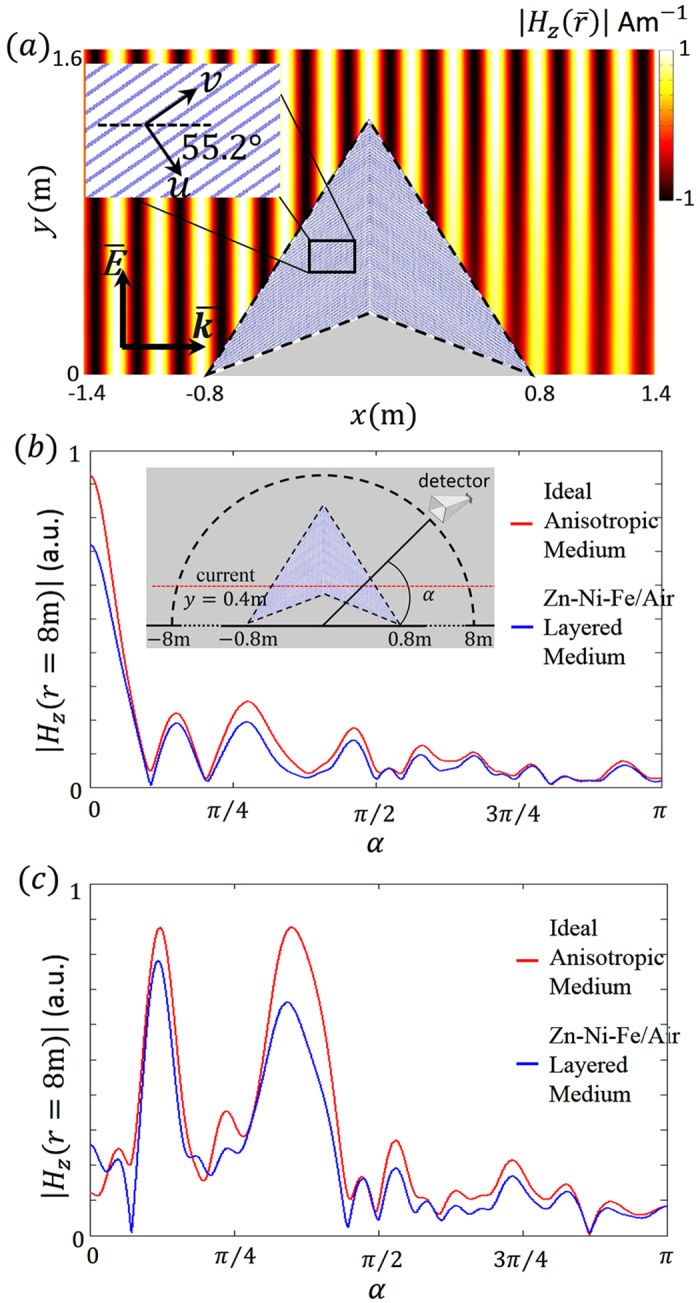
The designed carpet cloak with a layered structure and the scattering field excited by the spectral component of the current passing through it. **** (**a**) A plane electromagnetic wave with frequency 1 GHz passes the carpet cloak designed with Zn-Ni-Fe composite (blue) and air (white) in the surrounding background of Teflon. (**b**) The angular distribution of the scattering field along a semicircle far from the cloak excited by the current with frequency 1 GHz and phase velocity 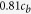. (**c**) The angular distribution of the scattering field along a semicircle far from the cloak excited by the current with frequency 1GHz and phase velocity 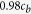.

To verify the results analyzed above, we propose a full-parameter carpet cloak that is implementable in reality. To facilitate observation, the radiation from the charged particle can be simulated in a microwave experiment by monitoring only one spectral component. It can be seen from equation (2) that the carpet cloak requires magnetism. Because of the 2D nature under consideration, we only need to construct three constitutive parameters: 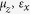 and 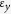. Simply using a layered metamaterial structure of homogeneous and isotropic materials, we design the carpet cloak as shown in Fig. 4a. The two materials that constitute the cloak are air with 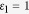 and 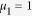 and Zn-Ni-Fe composite with 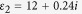
and 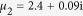
16 in the surrounding background Teflon with 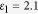 and 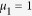. The thickness of air and the composite are 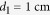 and 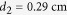 respectively. In the *u-v-z* orthogonal coordinates (*u*-axis and *v*-axis in the *x-y* plane; *u*-axis perpendicular to layers), the effective parameters are 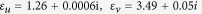
and 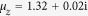. The angle between layers and the horizontal plane is 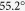. The left and right parts of the cloak can thus been constructed in a symmetric fashion. We simulate a plane wave at 1GHz with magnetic field perpendicular to the *x-y* plane going through the carpet cloak that is composed of realistic metamaterials as shown in Fig. 4a. The pattern on the right gets a little dim because of the loss from the carpet cloak.

We let the spectral component of the current (equation(4)) with frequency 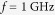 pass through the cloak. In the experiment, the spectral component of the current can be simulated by an antenna array with the phase velocity tuned by changing the uniform phase difference between two adjacent dipoles. This method has been used to study CR in metamaterials^17^,^18^. Fig. 4b,c show the angular distribution of the scattering field along the semicircle in the far field. In Fig. 4b, the phase velocity of the current is 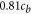. The red line represents the scattering field in a perfect scenario with an ideal anisotropic carpet cloak without any loss. The blue line represents the scattering field by the designed carpet cloak constituted of Zn-Ni-Fe composite and air in a background of Teflon. The profiles of the blue line and red line almost coincide, proving the effectiveness of the layered metamaterial structure. The small discrepancy mainly comes from the loss of the Zn-Ni-Fe composite. All the radiations come from TR for the relatively low phase velocity of the current. The strongest radiation occurs in the forward direction, which is also consistent with TR theory developed before^19^.

In Fig. 4c, the phase velocity of the current is 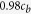, being faster than that in Fig. 4b The red line represents the scattering field in a perfect scenario with an ideal anisotropic carpet cloak. The blue line is from the designed carpet cloak with loss. The profiles coincide and the discrepancy mainly comes from the loss of the layered structure. It is interesting to see two peaks of radiation at about 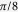 and 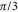. The radiation peak at 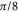 comes from the overlapped part of the upper branch of C1 and the lower branch of C2. The radiation peak at 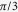 is mainly the combination of the lower branch of C1 and the upper branch of C2. These results are consistent with previous calculation shown in Fig. 3.

## Discussion

We analyzed in this paper the radiation generated by a moving charged particle passing through a perfect carpet cloak with uniform velocity. The radiation can reveal the asymmetry of transformation optics applied to motions of photons and charges. We proposed an experimentally verifiable model to demonstrate this asymmetry effect.

## Author Contributions

X.S., F.G. and X.L. performed the calculation and analyzed the results, B.Z. supervised the progress.

## Additional Information

**How to cite this article**: Shi, X. *et al.* Electromagnetic Detection of a Perfect Carpet Cloak. *Sci. Rep.* **5**, 10401; doi: 10.1038/srep10401 (2015).
